# LncRNA DLX6-AS1 as a potential molecular biomarker in the clinicopathology and prognosis of various cancers: a meta-analysis

**DOI:** 10.1042/BSR20193532

**Published:** 2020-08-25

**Authors:** Shubo Tian, Jinglei Liu, Shuai Kong, Lipan Peng

**Affiliations:** 1 Department of Gastrointestinal Surgery, Shandong Provincial Hospital Affiliated to Shandong First Medical University, Jinan, China; 2 Department of Gastrointestinal Surgery, Shandong Provincial Hospital Affiliated to Shandong University, Jinan, China

**Keywords:** DLX6-AS1, long-chain non-coding RNA, meta-analysis, tumor

## Abstract

*Objective*: Recent studies have shown that distal-less homeobox 6 antisense 1 (DLX6-AS1) is aberrantly expressed in various cancers and is associated with poor prognosis. This meta-analysis is designed to investigate the effects of DLX6-AS1 expression on clinicopathological features and survival outcomes. *Methods*: All eligible studies were searched from Pubmed, Web of Science, Embase, the Cochrane Library, and Wanfang database, up to August 2019. The literature was selected according to the inclusion and exclusion criteria listed in this work, and the quality of each eligible study was assessed. Each patient’s clinicopathological features and survival data were analyzed using Stata12.0 software. Begg’s test and sensitivity analysis were also conducted. *Results*: A total of 12 articles were included, covering 841 patients. Results showed that high expression of DLX6-AS1 was significantly closely associated with poor overall survival in tumor patients (hazard ratio (HR) = 2.30, confidence interval (95% CI): 1.70–3.09, *P*<0.01). This meta-analysis also showed that overexpression of DLX6-AS1 was significantly associated with tumor stage (*P*<0.01), tumor size (*P*<0.01), lymph node metastasis (*P*<0.01), and distant metastasis (*P*<0.01). Begg’s test suggested no publication bias. *Conclusion*: This meta-analysis revealed that high expression of DLX6-AS1 was related to the advanced clinicopathological characteristics of human digestive system cancers (gastric cancer, esophageal cancer, colon cancer, pancreatic cancer, and hepatocellular carcinoma) and other cancers such as ovarian cancer, osteosarcoma and non-small cell lung cancer, and DLX6-AS1 has important predictive value for poor prognosis. However, more studies are needed to further corroborate these findings.

## Introduction

Cancer is an important public health problem worldwide, and it is the leading cause of deaths in China. The development of cancer involves the ectopic movement, deletion, and expansion of chromosomes. Although the diagnosis and treatment of cancer have made great progress in recent years, the prognosis of cancer patients is still very poor. There is still a dearth of markers for early diagnosis of cancer and therapeutically effective targets.

Long-chain non-coding RNAs (lncRNAs) are a large class of RNA molecules that are over 200 nucleotides in length and relatively evolutionarily conserved. Although most lncRNAs cannot encode proteins, they participate in most cellular and physiological processes by acting as post-transcriptional regulators, enhancers, splicing regulators, and chromosomal modifiers. An increasing number of studies have shown that lncRNA is abnormally expressed in many human cancers, and it plays an important role in the initiation and progression of cancer. Previous meta-analyses have indicated that ZEB1-AS1 [1], MALAT1 [2], SNHG1 [3], DANCR [4], and HOTAIR [5] are related to the clinicopathological features and prognosis of tumors. These findings demonstrate that lncRNA may be a tumor-specific prognostic biomarker and therapeutic target [6].

Long non-coding RNA DLX6 antisense RNA 1 (DLX6-AS1), localized at the human chromosome 7q21.3, has been recently found to be tumor-related lncRNA. *DLX6* gene encodes a number of genes from the homeobox transcription factor family similar to the *Drosophila* distal-less gene. In humans, there are six distal-less homeobox (DLX) genes, represented by three double gene clusters, DLX1/DLX2, DLX5/DLX6, and DLX3/DLX4 [7]. Low expression of DLX genes has been observed in human solid tumors and hematological malignancies, suggesting that DLX genes play an important role in tumor growth and progression. More and more studies have reported that DLX6-AS1 is up-regulated in various tumor tissues, such as liver cancer, ovarian cancer, lung cancer, pancreatic cancer, and esophageal cancer and is associated with tumor invasion and metastasis. It has been confirmed that DLX6-AS1 functions as an oncogenic gene in several cancers acting as competing endogenous RNA (ceRNA). However, the role of DLX6-AS1 in cancer is still ambiguous. For example, Sun et al. reported that high expression of DLX6-AS1 was associated with tumor size and advanced clinical stage in non-small cell lung, but no significant associations between DLX6 expression and histological grade or lymph node metastasis [8]. In pancreatic cancer, high expression of DLX6-AS1 was positively correlated with large tumor size, advanced TNM stage, and lymph node metastasis [9]. Qian et al. indicated that DLX6-AS1 was overexpressed in gastric cancer tissues, it was positively associated with tumor size, lymph node involvement, and tumor staging [10]. Interestingly, Fu et al. found that overexpression of DLX6-AS1 was related to metastasis and advanced TNM stage, but was not related to tumor size and differentiation in gastric cancer [11]. Because these studies were limited by sample size and equivocal controversial. Therefore, we collected all relevant publications and conducted this meta-analysis to assess the correlation between DLX6-AS1 and clinicopathological features and prognosis in patients with malignant tumors.

## Materials and methods

### Search strategy and study selection

Eligible published literature was searched through Pubmed, Embase, Web of Science, Ovid, and Wanfang databases. The latest search time was 30 August 2019. The key words for search were ‘DLX6-AS1’ OR ‘distal-less homeobox6 antisense RNA 1’ OR ‘lncRNA DLX6-AS1’ AND ‘cancer’ OR ‘neoplasm’. The reference lists of included articles were also screened to avoid missing relative studies.

### Inclusion and exclusion criteria

The articles were considered eligible if they met the following criteria: (1) the relationship between DLX6-AS1 expression levels and clinicopathological features or survival in multiple human tumors were described; (2) studies that divided patients into high and low expression groups based on the expression levels of DLX6-AS1 in human tumor tissue; (3) DLX6-AS1 expression in patient tissues was measured by quantitative real-time polymerase chain reaction (qRT-PCR).

#### Exclusion criteria

(1) Studies that did not provide sufficient survival data or clinicopathological parameters; (2) letters, case reports, reviews, or animal studies; (3) studies from which necessary data could not be extracted or calculated from the original article.

### Data extraction

The data extraction was performed independently by two authors, and the relevant data were extracted according to the predesigned data extraction criteria. Disagreement was resolved through discussion or consultation with the third researcher. The data extracted from the included studies were first author’s name, year of publication, geographic region, tumor type, sample size, RNA detection method, follow-up time, type of survival analysis, hazard ratio (HR) and its confidence interval (95% CI), and the relationship between DLX6-AS1 expression and clinicopathological features of the patients. When the included literature had only survival curves, Engauge Digitizer software was used to obtain the survival information from the graphical plots using a previously described method [12,13]. When data are extracted, if the discussion does not yield results, an e-mail can be sent to the author to obtain the relevant data.

### Document quality evaluation

The quality of included literature was evaluated using Newcastle–Ottawa scale (NOS) standard, which included eight entries in three categories: selection, comparability, and outcome. Quality assessment scores ranged from 0 to 9 points, with scores above 6 points indicating better methodological quality. Quality assessment was carried out independently by two investigators and disagreements were resolved by discussion.

### Statistical processing

Statistical analyses were performed using Stata 14.0 software (Stata Corporation, TX, U.S.A.). For OS, we merged the HR values and conducted heterogeneity tests. The heterogeneity among different studies was determined using the chi-square-based Cochran *Q* test and *I^2^* statistic. *I^2^* > 50% was defined as significant heterogeneity. If there was no significant heterogeneity between studies, the fixed-effects model was used to analyze combined HR values and 95% CI. Otherwise, the random-effects model was used. HR > 1, indicated that patients with high DLX6-AS1 expression had poor prognosis. Pooled ORs and 95% CIs were used to estimate the relationship between DLX6-AS1 expression and clinicopathological characteristics including age, gender, tumor size, tumor stage, lymph node metastasis, and distant metastasis.

Publication bias was evaluated based on Begg’s funnel plots and Egger’s linear regression tests. *P*<0.05 was considered statistically significant. Sensitivity analysis was also performed by sequential omission of individual studies to assess stability of the results.

## Results

### Search results and basic characteristics of the included documents

**Figure 1:**
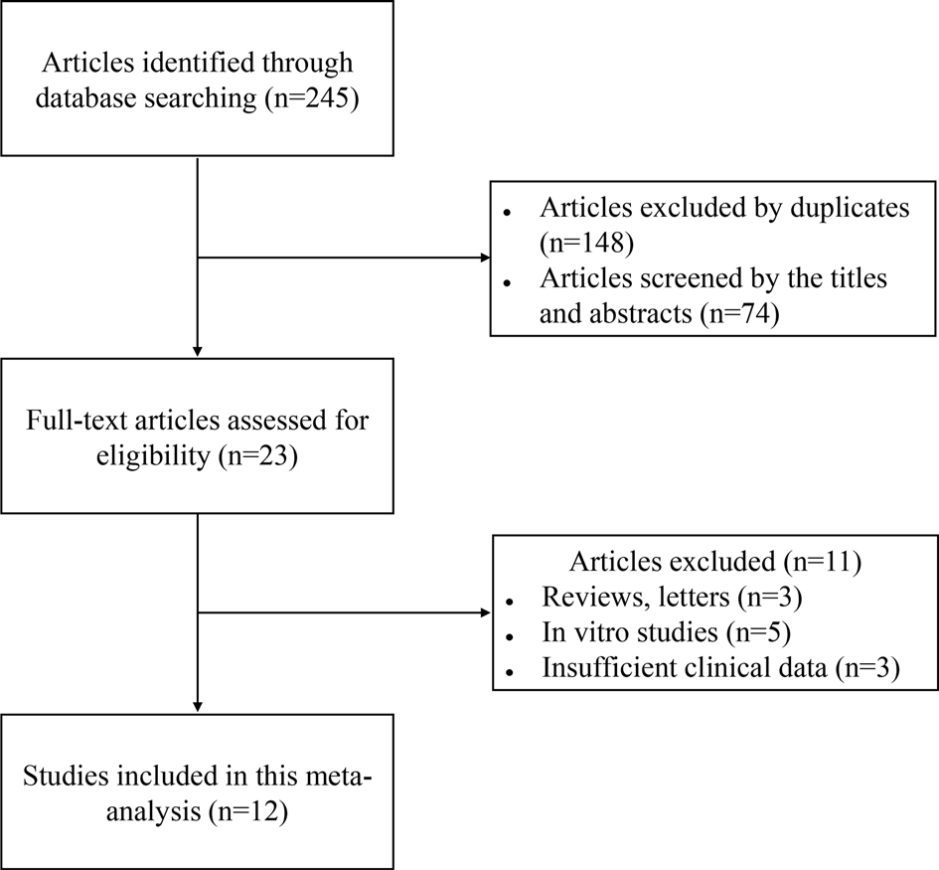
Flow diagram of the literature selection

According to the pre-established research strategy, the online databases were searched and the relevant works were obtained. The detailed screening procedure is shown in Figure 1.

A total of 245 articles were identified from the databases listed above. After exclusion of duplicate publications, 97 articles remained. Seventy-four studies were then excluded when their titles and abstracts were reviewed. A total of 23 articles were identified for full review, 11 of which were excluded due to lack of necessary data or *in vitro* studies. Finally, the remaining 12 articles [8,9,11,14–22] were included in this meta-analysis. These eligible studies were published between 2017 and 2019, and covered a total of 841 patients. The basic characteristics of the included studies are summarized in Table 1. The included studies addressed nine different tumor types: hepatocellular carcinoma (*n*=2), osteosarcoma (*n*=2), pancreatic cancer (*n*=2), ovarian cancer (*n*=1), non-small cell lung cancer (*n*=1), glioma (*n*=1), gastric cancer (*n*=1), esophageal cancer (*n*=1), and colon cancer (*n*=1).

**Table 1:** Basic information of included studies

Author	Year	Country	Tumor type	TNM stage	Sample size	Follow-up (months)	Survival analysis	Outcome measure	NOS
Dong-Mei Wu	2019	China	HCC	I–III	48	60	U	OS	8
Zhao J.	2019	China	OC	I–IV	128	60	U/M	OS/PFS	9
Rong-mou Zhang	2018	China	OST	I–IV	80	60	U/M	OS	6
Yong An	2018	China	PC	I–IV	84	NA	NA	NA	6
Wen Sun	2019	China	NSCLC	I–IV	51	NA	NA	NA	7
Ning Zhang	2018	China	OST	I–IV	20	NA	NA	NA	8
Xiangpan Li	2018	China	Glioma	I–IV	36	60	U	OS	8
Lei Zhang	2017	China	HCC	I–IV	60	60	U	OS	9
Jiyong Yang	2019	China	PC	NA	60	60	U	OS	8
Xiaodan Fu	2019	China	GC	I–IV	62	60	U	OS	8
Mingchen Wang	2019	China	EC	I–IV	73	NA	NA	NA	6
Wei Linlin	2019	China	CC	I–III	139	91	U/M	OS PFS	7

Abbreviations: CC, colon cancer; DFS, disease-free survival; EC, esophageal cancer; GC, gastric cancer; HCC, hepatocellular carcinoma; M, multivariate; NA, not available; NSCLC, non-small cell lung cancer; OC, ovarian cancer; OST, osteosarcoma; OS, overall survival; PC, pancreatic cancer; U, univariate.

### Basic information and quality evaluation of the included literature

A total of 12 studies were included, all performed on Chinese populations. DLX6-AS1 expression in these studies was detected using qRT-PCR in the patient’s tumor tissue, and the negative control was adjacent normal tissue. The quality of the included studies was evaluated using the NOS scale. All studies scored 6 points or more, indicating that these studies were of high quality (Table 1).

### Relationship between DLX6-AS1 expression and clinicopathological features

**Figure 2:**
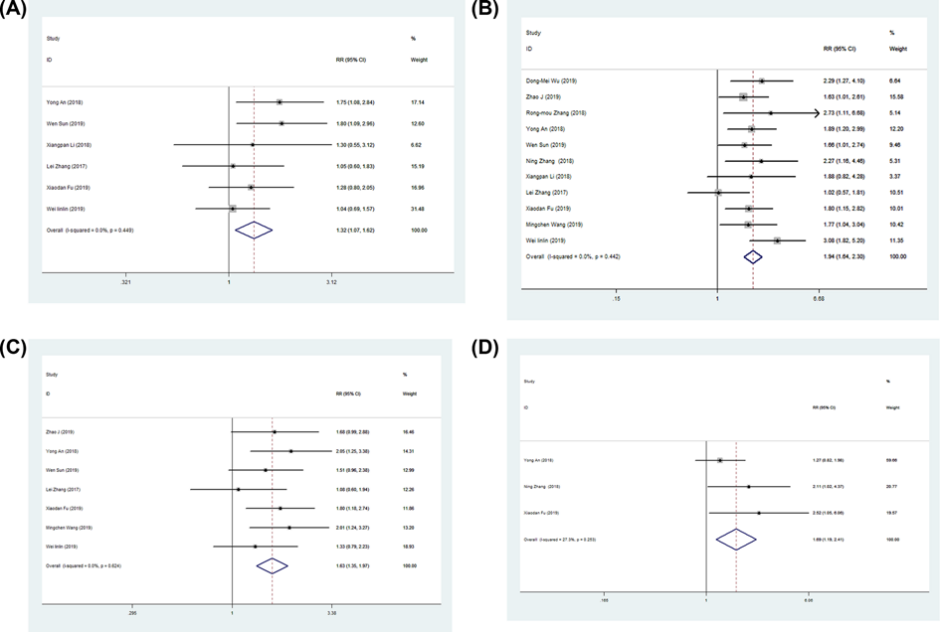
Forest plots of studies evaluating the relationship between DLX6-AS1 expression and clinicopathologic features Forest plots showing the association between DLX6-AS1 expression and tumor size (**A**), tumor stages (**B**), lymph node metastasis (**C**), and distant metastasis (**D**).

We evaluated the relationship between DLX6-AS1 expression and clinicopathological features in various cancers. As shown in Table 2, high expression of DLX6-AS1 was significantly associated with tumor size (OR: 1.32, 95% CI: 1.07–1.62; Figure 2A), tumor stages (OR: 1.94, 95% CI: 1.64–2.30; Figure 2B), lymph node metastasis (OR: 1.63, 95% CI: 1.35–1.97; Figure 2C) and distant metastasis (OR: 1.69, 95% CI: 1.19–2.41; Figure 2D). However, no significant association was found between DLX6-AS1 expression and age or gender in cancer patients. There was no significant heterogeneity among the studies (*P*>0.05, *I^2^* = 0 and 27.3%, Table 2), and thus the fixed-effects model was adopted.

**Table 2:** Meta-analysis for the association between DLX6-AS1 expression and clinicopathological parameters

Clinical parameters	Studies	Heterogeneity		Model	Meta-analysis	
		*I^2^*	*P*		OR (95% CI)	*P*
Age	11	0.00%	0.969	Fixed	1.07 (0.92, 1.24)	0.368
Gender	10	0.00%	0.855	Fixed	1.03 (0.87, 1.21)	0.731
Tumor size	6	0.00%	0.449	Fixed	1.32 (1.07, 1.62)	0.009
Tumor stage	11	0.00%	0.442	Fixed	1.94 (1.64, 2.30)	<0.001
Lymph node metastasis	7	0.00%	0.624	Fixed	1.63 (1.35, 1.97)	<0.001
Distant metastasis	3	27.3%	0.253	Fixed	1.69 (1.19, 2.41)	0.003

Abbreviations: OR, odds ratio.

In this way, the results indicated that high DLX6-AS1 expression significantly increased the risk of poor clinicopathological features.

### Association between DLX6-AS1 with survival time

**Figure 3:**
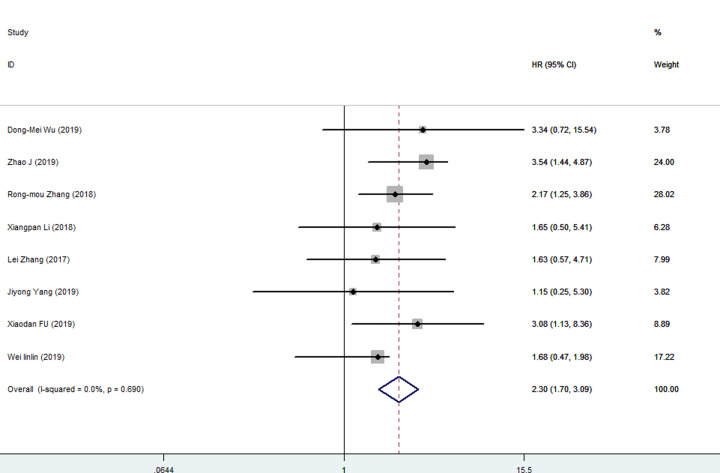
Forest plots for the meta-analysis of OS

A total of 8 studies with 613 patients were included in the meta-analysis of the association between DLX6-AS1 expression and overall survival in cancer patients. The results of *Q* test and *I^2^* test (*I^2^* = 0%, *P*=0.69) showed that there was no significant heterogeneity, so the fixed-effects model was used to estimate the pooled HR and 95% CI. The pooled HR indicated that higher DLX6-AS1 expression was related to worse survival (high DLX6-AS1 expression group versus low DLX6-AS1 expression; pooled HR = 2.30, 95% CI: 1.70–3.09, *P*<0.01, Figure 3), which demonstrated that DLX6-AS1 was a risk factor for the prognosis of cancer patients.

### Publication bias and sensitivity analysis

**Figure 4:**
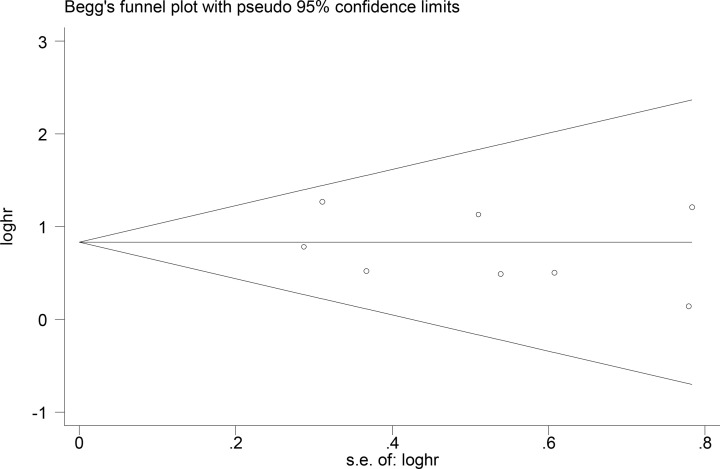
Funnel plot for the meta-analysis of OS

Begg’s funnel plot and Egger’s test were conducted to evaluate the publication bias of these studies. The results showed that the funnel plot scatters symmetrically, and there was no obvious publication bias (Begg’s test: *P*=0.711; Egger’s test: *P*=0.447; the difference is not statistically significant, Figure 4).

**Figure 5:**
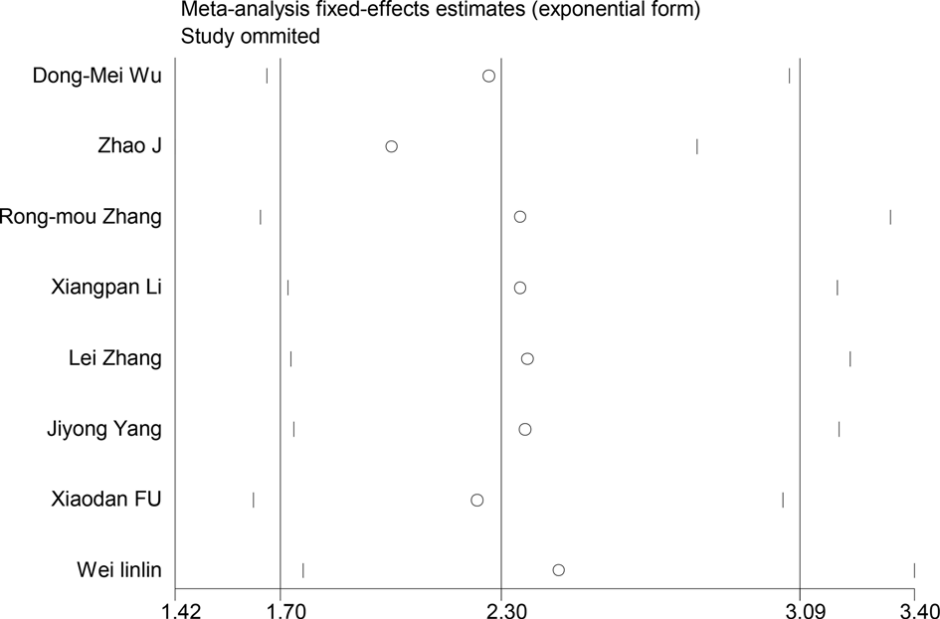
Sensitivity analysis for the meta-analysis of OS

Sensitivity analysis was performed to estimate the effect of each study on the overall results. It revealed that the pooled DLX6-AS1 HR was not significantly affected by the exclusion of any single study. This suggests that the results of the combined analysis are better and more stable, as shown in Figure 5.

## Discussion

LncRNAs play key roles in gene expression and are involved in tumor development, occurrence, and prognosis [23]. Accumulating lines of evidence have proven that lncRNAs could be targets for cancer diagnosis and may be suitable biomarkers, such as HOTAIR [5], H19 [24], and XIST [25]. DLX6-AS1 is the antisense transcript of the *DLX6* gene, which is a newly discovered lncRNA and has been confirmed as up-regulated oncogene in tumorigenesis. Several studies have suggested that DLX6-AS1 knockdown significantly attenuated cell motility, proliferation, and invasion. However, the mechanisms underlying the role of DLX6-AS1 in mediating tumor metastasis have not been elucidated.

LncRNAs can act as miRNA sponges, reducing their regulatory effect of mRNAs. In ovarian cancer, DLX6-AS1 can regulate the progression of ovarian cancer by sponging miR-613 [26]. DLX6-AS1 has also been proved to serve as a ceRNA to alter the expression of target miRNAs. In pancreatic cancer, DLX6-AS1 has been found to up-regulate the expression of Zinc finger E-box-binding homeobox 2 (ZEB2) by sponging miR-181b and then promote cancer cell proliferation and invasion [9]. A study by Li et al. found that DLX6-AS1 modulates gliomagenesis by competing endogenous sponging miR-197-5p to relieve E2F1 [18]. Knockdown of DLX6-AS1 suppressed the growth and invasive abilities of cancer cells by disrupting the epithelial–mesenchymal transition program in gastric cancer [11,27] and esophageal squamous cell carcinoma [28]. DLX6-AS1 was found to promote tumorigenesis through the NOTCH [15], Wnt [16], and PTEN [29] signaling pathway. All these findings indicated that lncRNA DLX6-AS1 could function as an oncogene and predict poor prognosis in cancer patients. However, these studies had small sample size and lacked systematic research. Our meta-analysis reviewed the published articles on DLX6-AS1 expression and clinicopathologic characteristics and prognosis in various cancers. As far as we know, this is the first meta-analysis to comprehensively assess the relationship of DLX6-AS1 expression with prognosis and clinicopathologic features in various cancers. The results showed that high expression DLX6-AS1 was significantly closely correlated with poor outcomes in patients with various types of cancers. Patients expressing high levels of DLX6-AS1 were associated with lower survival rates than those with low expression. Our results also revealed that increased DLX6-AS1 expression was associated with tumor TNM stage, lymph node metastasis, and distant metastasis, which suggest that increased DLX6-AS1 may be associated with advanced features of cancer.

Although the present study has a strict search strategy and inclusion and exclusion criteria, there are still some unavoidable limitations. First, some HR values were not given in the literature and could only be calculated by extracting the data from the survival curve, so the values may not be fully accurate. Second, after searching multiple databases, 12 articles were included here. The total sample size was relatively small, which might reduce the statistical effect of the combined effect. Third, there were differences in the cut-off value of DLX6-AS1 expression among the included studies. This will increase the difficulty of using DLX6-AS1 as a clinical prognostic marker. Finally, most patients included in the study were from China, which may lead to geographical bias. So, well-designed multicenter studies in different populations are warranted to achieve a more persuasive conclusion.

## Conclusion

In summary, our study suggests that high levels of DLX6-AS1 expression are a valuable predictor of poor cancer prognosis in overall survival, TNM stage, tumor size, lymph node metastasis, and distant metastasis. Expression of lncRNA DLX6-AS1 is not associated with age or gender, so, despite the limitations listed above, the meta-analysis allows us to draw the conclusion that DLX6-AS1 may be a novel prognostic molecular marker for several types of cancers in Chinese population.

## Abbreviations

ceRNA: competing endogenous RNA
DLX: distal-less homeobox
DLX6-AS1: distal-less homeobox 6 antisense 1
HR: hazard ratio
lncRNA: long-chain non-coding RNA
NOS: Newcastle–Ottawa scale
OS: overall survival
qRT-PCR: quantitative real-time polymerase chain reaction
TNM: Tumor, Node and Metastasis
95% CI: confidence interval
